# Nondestructive inspection of surface nanostructuring using label-free optical super-resolution imaging

**DOI:** 10.1038/s41598-023-32735-w

**Published:** 2023-04-12

**Authors:** Alberto Aguilar, Alain Abou Khalil, David Pallares Aldeiturriaga, Xxx Sedao, Cyril Mauclair, Pierre Bon

**Affiliations:** 1 Xlim Research Institute, CNRS UMR 7252, Universitéde Limoges, Limoges, France; 2 UMR 5516 CNRS, Hubert-Curien Laboratory, University of Lyon, Jean-Monnet University, 42000 Saint-Etienne, France; 3 GIE Manutech-USD, 42000 Saint-Etienne, France

**Keywords:** Ultrafast lasers, Super-resolution microscopy, Surface patterning, Laser material processing, Confocal microscopy

## Abstract

Ultrafast laser processing can induce surface nanostructurating (SNS) in most materials with dimensions close to the irradiation laser wavelength. In-situ SNS characterization could be key for laser parameter’s fine-tuning, essential for the generation of complex and/or hybrid nanostructures. Laser Induced Periodic Surface Structures (LIPSS) created in the ultra-violet (UV) range generate the most fascinating effects. They are however highly challenging to characterize in a non-destructive manner since their dimensions can be as small as 100 nm. Conventional optical imaging methods are indeed limited by diffraction to a resolution of $\approx 150$ nm. Although optical super-resolution techniques can go beyond the diffraction limit, which in theory allows the visualization of LIPSS, most super-resolution methods require the presence of small probes (such as fluorophores) which modifies the sample and is usually incompatible with a direct surface inspection. In this paper, we demonstrate that a modified label-free Confocal Reflectance Microscope (CRM) in a photon reassignment regime (also called re-scan microscopy) can detect sub-diffraction limit LIPSS. SNS generated on a titanium sample irradiated with a $\lambda =257$ nm femtosecond UV-laser were characterized with nanostructuring period ranging from 105 to 172 nm. Our label-free, non-destructive optical surface inspection was done at 180 $\upmu$m$^2$/s, and the results are compared with commercial SEM showing the metrological efficiency of our approach.

## Introduction

Laser ultra-high precision processing of materials has been widely known for more than five decades. More specifically, the generation of Laser-Induced Periodic Structures (LIPSS or ripples) is a universal phenomenon paving the way for numerous applications^1^, such as tuning the wettability of surfaces^2,3^, adding anti-bacterial functionalities^2,4^, or controlling cell adhesion^5–8^. Femtosecond lasers can generate LIPSS on surfaces with a periodicity that depends on the irradiation wavelength. Two types of LIPSS have been reported in the literature^9^: Low Spatial Frequency LIPSS (LSFL) and High Spatial Frequency LIPSS (HSFL). When the LSFL exhibit a period $\Lambda _L$ larger than half of the laser irradiation wavelength $\lambda _i/2$, generally oriented perpendicular to the laser polarization, the HSFL show a periodicity $\Lambda _H$ lower than half of the laser irradiation wavelength. Using shorter irradiation wavelength results in lower ripple periodicity, for both LSFL and HSFL, and recent cutting-edge developments in ultraviolet (UV) fs lasers unlock sharper surface structuring with enhanced optical, chemical or mechanical effects^3,10–12^. To characterize these nanostructures Scanning Electron Microscopy (SEM) and Atomic Force Microscopy (AFM) are the gold standard tools but are challenging on dielectric or fragile samples that cannot be metalized or when LIPSS amplitude is of interest. Moreover, these characterization methods (SEM and AFM) for nano-structures (such as LIPSS) are, in general, performed off-site at a remote location (to the laser patterning setup) dedicated to the characterization instruments. In-situ, off-site analysis is possible but this often requires tailor-made integration of laser facilities and analysis instruments^13^. An optical system that enables the metrological study of small objects such as small LIPSS would accelerate the parametric study and boost the research pace with a cost-effective solution. Nondestructive inspection of large period LIPSS -generated for instance with an infrared (IR) fs laser- could be envisioned to be characterized by conventional optical imaging methods. Indeed, LSFL have periodicity around 600 nm, and HSFL readily below 300 nm, close to the best resolution limits, thus hardly contrasted. However, visible or UV fs lasers generate HSLF and LFSL LIPSS with periodicity falling below the optical diffraction limit : to keep nondestructive inspection regime, optical imaging methods that overcome this diffraction limit are thus essential.

Super-resolution microscopy (SRM) has been formalized in the 90’s and has been an emerging tool in several areas, especially biology and medicine^14–17^. Most SRM techniques like STORM, STED, or nonlinear SIM require labeling (usually with fluorophores) and are exploiting fluorescence photo-physics to surpass the diffraction limit and reach over 50 nm of lateral resolution^16,18–20^. However, for material sciences, samples like glasses, metals, or polymers—where surface functionalization with fluorescence is not an easy task (when possible)^21^- the use of SRM remains limited. Label-free super-resolution optical system has few implementation in the literature. For instance, 50 nm resolution can be obtained using the virtual image created by microspheres placed over the sample to collect near-field information from the analyzed surface^22^. It remains however challenging to perform metrological measurements in-situ, where an extra element like a microsphere would change the integrity of the material. Some indirect optical imaging methods has been implemented to characterize periodic surfaces by observing the diffraction pattern caused by the back-scattered light. But artifacts may arise when the periodicity breaks or the initial structure orientation is changed^23,24^. Structured illumination have been used as an alternative to increase the modulation transfer function of the microscope and thus the image contrast on non-fluorescent periodic samples. It is a good alternative for *in-situ* measurements^25^. However, this technique requires a sophisticated algorithm to retrieve the image and thus is not a *what-you-see-is-what-you-get* method, with potential reconstruction errors.

To increase the spatial resolution of the microscope, we propose to use a label-free confocal reflectance microscope (CRM) in a photon-reassignment regime^26^. One of the main characteristics of photon-reassignment CRM, also known as re-scan CRM, is to sample the image point spread functions (PSFs) on a matrix sensor^27^ rather than just counting the photons on a single-pixel detector (photodiode or photomultiplier) as it is commonly done in conventional-CRM. The photon-reassigned principle is based on the reallocation of the detected photons by shrinking the image PSF. By giving more distance between the adjacent PSFs, higher-frequency information becomes available. The frequency support can be up to doubled^26,28^ with re-scan RCM as compared to conventional-RCM, leading *in fine* to a resolution gain lying between 1.4 and 2 (after frequency domain re-weighting, a type of deconvolution)^29^ ; moreover, the setup can be adapted to a fully optical configuration^30^, minimizing the computing time. Re-scanned CRM offers a label-free high-resolution image, that neither requires *a priori* information on the sample, such as the surface characteristics and material nature, nor control of light polarization, which might be altered by LIPSS. It has been previously reported how the technique can achieve a 90 nm lateral resolution, for biological samples and nanomaterials^31^. Moreover, since super-resolution is obtained with back-scattered light, the acquisition speed is only bounded by the speed of the scanners, which can be useful for potential *in situ* measurements while structuring the surface, with the possibility of additional measurements such as Raman imaging to retrieve molecular information^32^, or optical profilometer (see SI).

In this paper, we perform metrological characterizations of nanometer UV-generated LIPSS (periodicity in the range of 105 - 172 nm) using our full-optical label-free method based on photon reassignment. We compare the method to conventional CRM and non-optical gold standard methods (SEM and AFM). These results, pave the way toward an *in-situ*, label-free, cost-effective inspecting of any SNS.

## Results

### Characterizaton of LIPSS with SEM

**Figure 1:**
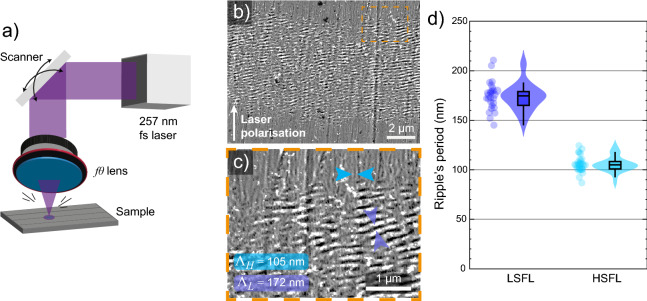
SEM images of generated LIPSS with a 257 nm UV fs-laser. The zoomed image reveals a periodicity ($\Lambda$) arround 105 and 172 nm respectively for HSFLs and LSFLs. (**a**) Femtosecond laser setup for LIPSS formation, (**b**) SEM observed area with a 11,000$\times$ magnification, (**c**) 30,000$\times$ magnification for HSFL and LSFL visualization, (**d**) Measured distribution of the LSFL and HSFL period $\Lambda _L=172$ nm, $\sigma _L=14$ nm, $\Lambda _H=105$ nm, $\sigma _H=5$ nm.

LIPSS were obtained over a titanium alloy sample, using a fourth harmonic UV femtosecond laser ($\lambda _i=257$ nm, see “Methods” for details). The nanostructures were created on a setup dedicated to the LIPSS formation (Fig 1a) and then analyzed on the re-scanned CRM setup. The generated LSFL and HSFL were observed with a SEM on a textured area of 40 $\upmu \text {m}^2$ finding respectively a period $\Lambda _{\text {L}}=172\pm 14$ nm and $\Lambda _{\text {H}}=105\pm 5$ nm, as shown in Fig 1b–d.

The dimensions of these structures fall close or smaller than the best optical resolution limit: $\lambda /(2\cdot NA) \ge 154$-nm, with $NA\le 1.3$ (water immersion objectives to keep non-destructive optical characterization) and $\lambda \ge 400$-nm to allow the use of such objectives. Moreover, the image content is not only ruled by the resolution limit: the modulation transfer function (MTF)^33^ -which characterizes the contrast as a function of the object frequency- is vanishing to zero when approaching the system cutoff frequency. Although theoretically resolved, even LSFL are challenging to observe.

### LSFL optical imaging

**Figure 2:**
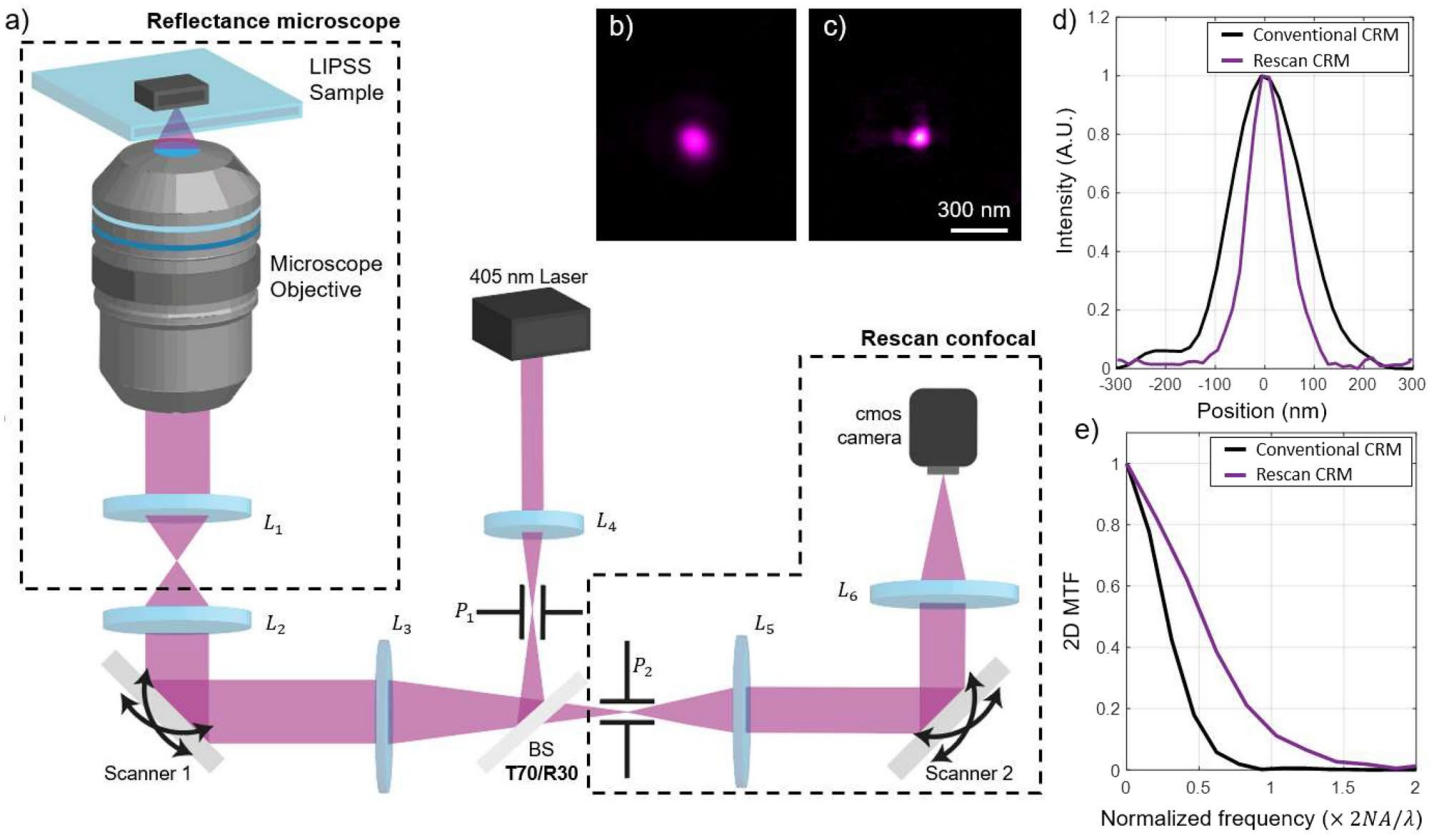
Optical imaging setup and resolution of the system. (**a**) Schematic of the optical setup, (**b**) conventional reflectance confocal microscope PSF, (**c**) re-scanned confocal microscope PSF, (**d**) radial average of the PSFs shown on (**b,c**), (**e**) modulation transfer function of the system.

Using a 405 nm continuous laser and a $NA=1.27$ microscope objective assembled in the setup of Fig. 2a, we obtained a resolution of 176 nm (theoretical resolution limit: $r_o=160$ nm) with regular CRM; when moving to super-resolution mode with our re-scan CRM, the lateral resolution was measured at $r_{\text {rs}}=92$ nm, as observed in Fig. 2d using the full width at half maximum of the PSF. This is a resolution gain of 1.91, enough to inspect both LSFL and HSFL. It is important to mention that this is only an estimation of the resolution since we are working in a coherent imaging regime^34^. In the Fig. 4, we demonstrate on actual quasi-periodic structures that the resolution is at least 105 nm.

**Figure 3:**
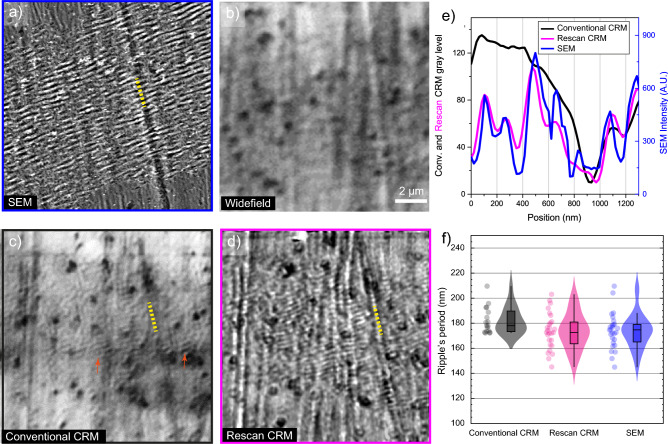
LSFL over titanium alloy surface created by fs laser ($\lambda _i=257$ nm) and analyzed with different imaging techniques. (**a**) SEM, (**b**) widefield imaging, (**c**) conventional CRM, the orange arrows indicates visible LIPSS of period >180 nm, (**d**) re-scan CRM, (**e**) plot of the transversal cut shown in dashed white in (**b**–**d**), (**f**) LSFL period distribution with the 3 imaging methods.

Inscribed LSFL have a period between 145 and 190 nm (Fig. 3a). No ripples were detected with widefield imaging (Fig. 3b), only a few are detected with conventional CRM (Fig. 3c) while super-resolved re-scan CRM unlocks a clear visualization of the ripples (Fig. 3d).

First, re-scan CRM increments the lateral resolution almost twice as the conventional CRM (Fig. 2e) and ripples with dimension smaller than the diffraction limit are now detected. Second, the contrast of the re-scan CRM image is enhanced as compared to conventional CRM (i.e. higher MTF) improving the detection efficiency of the already resolved ripples.

A transversal cut on SEM, CRM, and re-scan CRM images (Fig. 3a,c,d) is plotted and displayed in Fig. 3e. The analysis shows how ripples around the resolution limit are impossible to distinguish for a conventional CRM, but -when compared to SEM-the CRM re-scanned version shows similar intensity variations with congruent period.

The period of the LSFL was analyzed in an area of 40 $\upmu \text {m}^2$ of the nanotextured surface using SEM, conventional CRM, and re-scan CRM. The distribution of the measured period through the treated surface for the three different methods is shown in Fig. 3f. For the conventional CRM, the measured average period of LSFL was 187 nm, with a standard deviation of 12 nm, meanwhile, for both SEM and super-resolved re-scan CRM, the average period was 172 nm, with a standard deviation of 14 nm. The measurement discrepancy with conventional RCM arises from a biased measurement due to the resolution limit threshold that discards the smallest ripples.

### HSFL visualization

**Figure 4:**
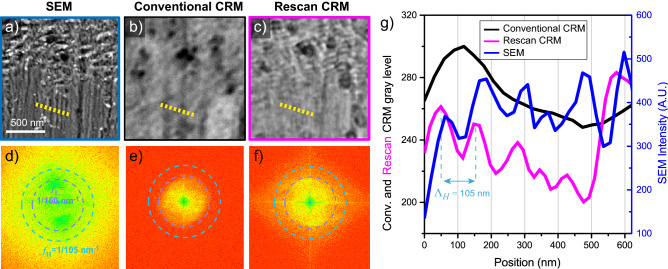
HSFL over titanium alloy surface created by fs laser ($\lambda _i=257$ nm) and analyzed with different imaging techniques, including their frequency representation. (**a**) SEM, (**b**) conventional CRM, (**c**) re-scan CRM, (**d**) frequency representation of (**a**), (**e**) frequency representation of (**b**), (**f**) frequency representation of (**c**), (**g**) plot of the transversal cut shown in (**a–c**).

The period of the HSFL ($110 \pm 5$-nm), is beyond the cut-off frequency of any regular optical microscope. The MTF of the re-scan CRM stops at 92 nm, at the limit to observe the HSFL in the sample. Fig. 4a shows the structured surface imaged with the SEM and is compared to regular CRM and re-scan CRM (Fig. 4b,c). Even though the contrast is not as high as in the LSFL (Fig. 3d), it is sufficient to distinguish the HSFL with re-scan CRM and a few high-frequency ripples remain undetected. A transversal cut of the marked area in Fig. 4a–c is plotted in Fig. 4g, showing that the characterization of HSFL with re-scan CRM is possible (see SI for more details).

The frequency support for all the techniques was computed using the Fourier transformation of the images (Fig. 4a–c). The frequency support of the SEM easily includes nanometre information, as expected; a circle with pointed blue dots and a orange one show respectively the spatial frequency corresponding to the LSFL mean period, and the HSFL mean period (Fig. 4d). The comparison with the conventional CRM frequency support (Fig. 4e) confirms that the information support does not even include the LSFL cut-off frequency. Thus, for this sample region, even the LSFL can not be inspected with the conventional CRM. On the other hand, the re-scan CRM support (Fig. 4f) goes up to the HSFL cut-off frequency, so both LSFL and HSFL can be analyzed using our super-resolved technique.

## Discussion

Beyond these unprecedented ripple’s nondestructive optical inspection, our nanometre surface analysis method exhibits other key advantages. Being based on an optical microscopy setup, re-scan CRM benefits from the non-contact flexibility of the technique. This sub-100 nm label-free resolution is particularly adapted to detect and analyze laser surface structuring with short and ultrashort pulses, since this structuring occurs commonly at the submicrometric scale. When compared to gold standard metrological methods such as AFM or SEM, re-scan CRM has strong advantages including that (i) contact-less direct acquisitions on unaltered sample are possible (i.e. no vacuum, tips or metalization are required). (ii) The acquisition speed is currently of 0.8 s to inspect a 12 $\upmu$m by 12 $\upmu$m area, so 180 $\upmu$m$^2/s$, and could be speed up to 1 mm$^2$/s by moving to resonant scanning. (iii) The elegant simplicity of our optical characterisation setup makes it a cost-effective, easy to deploy and maintain method. Moreover, the acquisition pace of re-scan CRM has the potential to be performed in-situ, during the laser processing. The image does not require numerical treatment as all the process is done optically by the two mirrors. For maximal contrast and resolution, photon re-weighting via deconvolution is strongly suggested after the acquisition, see SI for discussion and comparison. The CRM and re-scan CRM images (and extracted profiles) presented in this paper are deconvolved images. Noteworthy, quantitative ripples height extraction from the deconvolved signal is not direct since the deconvolution can only be performed on the intensity image and not the actual electromagnetic field.

To image a similar region, it would take about 10 min with a conventional AFM, not taking into account the user-dependant time for sample manipulation, fine positioning under the AFM to find the area of interest, that are required for this ex-situ analysis. Concerning SEM, it can largely surpass that of re-scan CRM in terms of resolution since it is not limited by optical diffraction. Its acquisition time is in the range of seconds for the same measured area. However, the indispensable controlled environment (like a certain level of vacuum) induces time-consuming sample manipulation and makes this methods incompatible with in situ characterization.

Super-resolved re-scanned CRM has the capability to perform in situ inspection which can address two major current challenges in LIPSS patterning. Firstly, in-situ metrological measurements are mandatory to assess the stability of the ripples regardless of the local material properties^25^. Secondly, especially for ultrafast laser, a real time inspection feedback loop drastically boosts the speed and precision of the laser multiparameter’s optimization, including the peak power, repetition rate, pulse duration, polarization, spatial overlap but also the temporal and spatial beam intensity distribution. For example, the major field of laser welding has known a change of paradigm when in-situ monitoring of the keyhole^35^ has been made possible. Furthermore, in-situ optical characterization technique has been used to optimize the ultrafast laser temporal shape for bulk photo inscription in borosilicate glass^36^ or to match a user-defined surface ablation pattern^37^. Re-scan CRM unlocks for the first time such optimizations at the nanometre scale where several surface functions can be achieved with notably biomedical interest and complex hybrid surfaces.

## Methods

### Optical setup

Optical super-resolution was obtained using a confocal reflectance microscope based on photon reassignment, as observed in Fig. 2a. A 405 nm continuous laser (L405G1, Thorlabs, USA) was used as an illumination source; the laser was spatially filtered by a pinhole ($P1= 50 \upmu$m) to be perfectly diffraction limited and then directed to a homemade microscope by a *T*70/*R*30 beam splitter (*BS*). The light is projected into a 2-axis galvo mirror (ScannerMAX Compact 506 Scanners, Pangolin, USA) by the lens $L_3$ ($f=200$ mm). This mirror is in charge of scanning the laser over the sample, just like any conventional confocal microscope.

To get the image, a microscope objective ($60\times$, water immersion, NA $=$ 1.26, Nikon, Japan) focuses the light on the sample and collects the light back-scattered by the analyzed sample. This back-scattered light is descanned by the same galvo mirror, and focused on a second pinhole ($P_2=300 \, \upmu$m), where only the light that is in focus can pass through the pinhole. The light is collimated by a lens ($L_5=300$ mm) and projected onto a second *x*–*y* scanner which oversees the back-scattered photon reassignement to increase by two fold the separation between two adjacent PSFs. Light is finally focused on a CMOS camera (DCC1545M, Thorlabs, USA) by lens $L_6=200$ mm. The result is a direct super-resolution image on the CCD. The average speed of the galvo mirror for small angles is approximately 1 kHz; the mirrors were controlled using an ADC/DAC acquisition card (NIUSB-653, National Instrument, USA). All the process to reach a super-resolution regime is done optically by the two scanners but image deconvolution can be applied after the optical reconstruction to maximize the resolution gain and image contrast (see SI). Figure 2b shows the PSF of a conventional confocal reflectance microscope, which can be compared directly with the PSF of the re-scanned CRM, shown in Fig. 2c. Both PSFs were obtained by the setup shown in Fig. 2a, with a sample of 60 nm diameter gold nanoparticles (OD 1, stabilized suspension in citrate buffer, PubChem Substance ID 329765549, Sigma Aldrich) deposited on a type 1.5 coverslip. The degradation of the experimental PSFs is because of optical aberrations (mostly coma and astigmatism). These aberrations are caused by the optical quality of the surface of the galvo-mirrors, which are only guaranty at $\lambda /2.5$ at $\lambda =405$ nm.

### LIPSS generation

LIPSS formation was performed with a femtosecond laser (PHAROS, Light conversion, Lithuania) working at 50 kHz with 160 fs pulse duration and 60 nJ pulse energy. The fourth harmonic (257 nm) is achieved with HIRO Harmonic generator from the same company. The scanning was performed with a galvo scanner (Thorlabs) and an 100 mm f-$\theta$ lens. Lines were written at a speed of 20 mm/s (98.4% overlap ratio). The sample was a titanium alloy (Ti6A14V, Goodfellow) with an initial arithmetical mean high (Sa) of 0.052 $\upmu$m.

After an ultrasonic bath, the sample was inspected with a commercial SEM (Jeol, Japan). Figure 1a depicts the laser line inscription. LIPSS are constituted of LSFLs distributed parallel to the inscription direction and perpendicular to the laser polarization (found at the center); and HSFLs arranged perpendicular to the scanning direction and parallel to the laser polarization (observed at the borders of the inscription).

## Supplementary Information

Supplementary Information.

## Data Availability

The datasets used and analysed during the current study available from the corresponding author on reasonable request.
