# Supplementary material for: Nondestructive inspection of surface nanostructuring using label-free optical super-resolution imaging
Source: Sci Rep. 2023 Apr 12;13:6008. doi: 10.1038/s41598-023-32735-w (PMC10097710; doi:10.1038/s41598-023-32735-w)
Supplement: Supplementary file 1 — Supplementary Information. [file 41598_2023_32735_MOESM1_ESM.pdf]

Supplementary information: Nondestructive inspection of surface nanostructuring using label-free optical super-resolution imaging

by: Alberto Aguilar, Alain Abou Khalil, David Pallares Aldeiturriaga, Xxx Sedao, Cyril Mauclair, Pierre Bon

Optical Phase profilometry

Re-scan optical microscopy can be easily used jointly with another optical measurement that would give extra information for surface characterization, such as Raman emission or phase measurement for surface profilometry. Suppl. Fig. 1 shows how the phase delay introduced by the surface can be measured while the super-resolution surface inspection is done. The imaging system is the same as presented for LIPSS measurements; however, a quantitative phase imaging stage based on a quadriwave lateral shearing interferometer[1] is placed in the path and the sample is illuminated in widefield using a 405-nm LED.

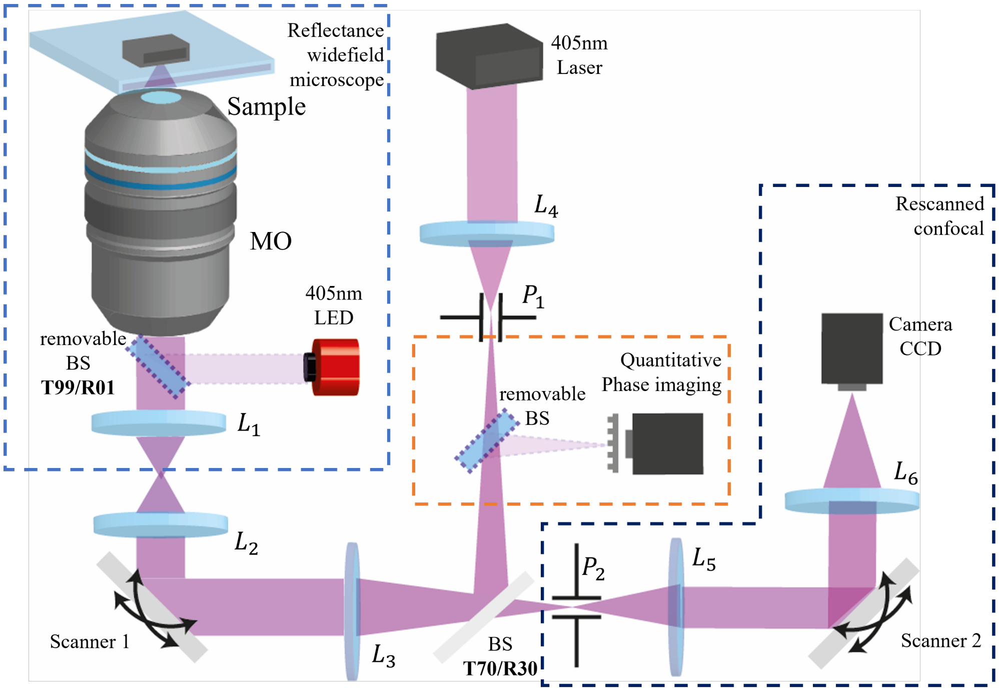

Figure 1. Optical setup for surface inspection using both photon reassignment microscopy and widefield quantitative phase imaging for profilometry.

Results can be observed in Fig. 2. Where LIPSS can be easily observed using the re-scan CRM technique (Fig. 2.a), with a lateral resolution beyond the diffraction limit, the complementary measurement of the light phase gives a direct evaluation of the surface elevation (suppl. Fig. 2.b). Results were compared with an atomic force microscope, Fig 2c, (CSI Nano-Observer, Shaefer, Germany). A transversal cut from suppl. Fig.2.b-c is analyzed and plotted (suppl. Fig2 d) in order to compare the data.

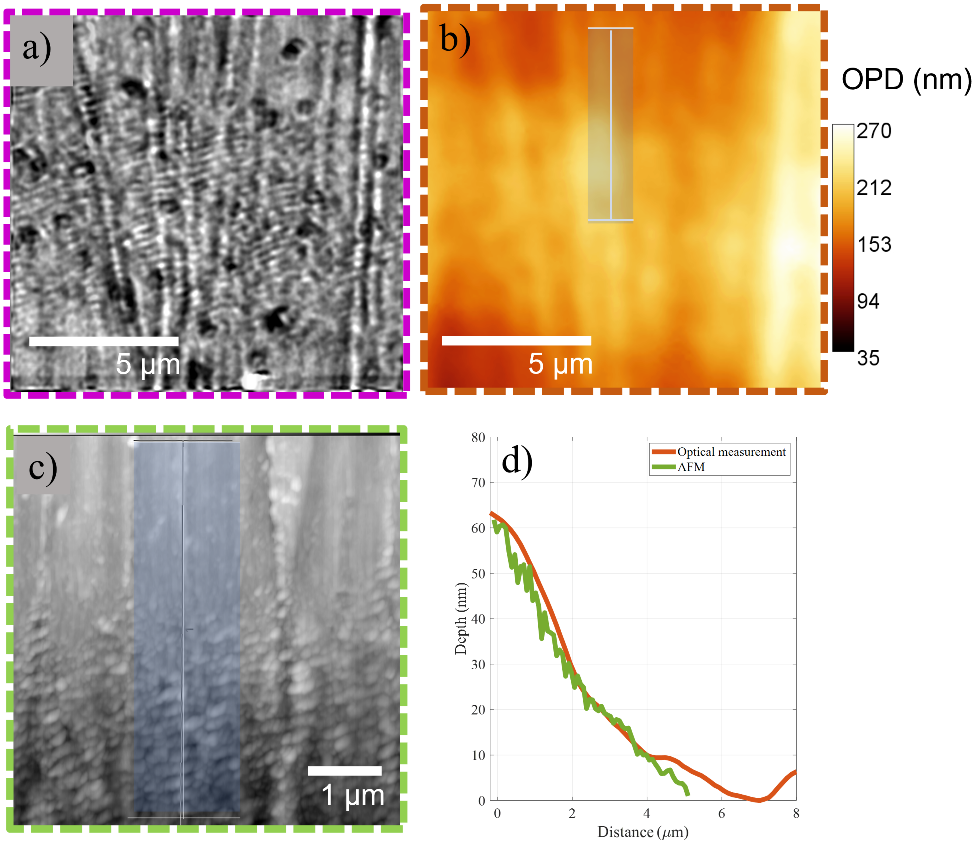

Figure 2. Quantitative measurements of the laser-processed surface. a) Re-scan CRM, b) Quantitative optical path difference measurement of the surface observed in reflection, c) AFM image of the surface, d) plot of the transversal cut shown in *b)* (converted in height), and *c)*.

HSFL period measurement

HSFL have sizes within the limit of the MTF of the re-scan CRM which leads to a limited contrast. To track the period of the ripples, a segmentation algorithm with a Kalman filter was implemented. The Kalman filter was restricted in the areas where the HSFL ripples are visible, with an orientation research close 90 degree as compared to the orientation of the LSFL (kwnowing the laser polarization). This helps to isolate and discriminate the ripples from the surface scratch. A second restriction is implemented in the filter, to only recognize the structures that shows a mean period of 100 ± 20 nm. The calibration of the filter was done over the SEM images (suppl. Fig. 3.a) and then implemented on the CRM Re-scan image (suppl. Fig. 3.c). An image of the binarized filter is displayed over the image for representation, as shown in suppl. Fig. 3.b,d.

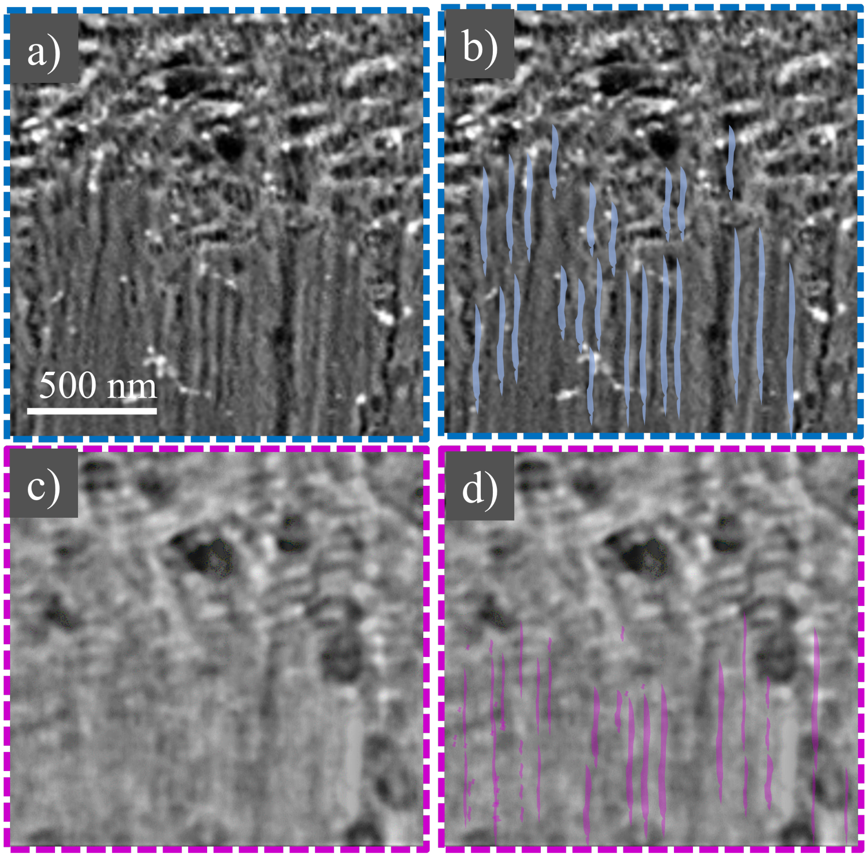

Figure 3. **HSFL detection**, a) SEM image, b) composite binarized filter over the SEM image, c) re-scan CRM image, d) composite binarized filter over the re-scan CRM image

Image deconvolution

Re-scan CRM technique generates a single image, like in wide-field microscopy. There is no need for extra numerical reconstruction to retrieve an image. However, since the recorded image is convolved by the detection PSF, a deconvolution procedure (also called photon reweighting for photon reassignment methods[2]) is required to enhance the contrast of the image and highlight the smallest features in the images. Suppl. Fig. 4.a shows the raw image generated by the re-scan CRM technique. Using a classic Lucy-Richardson deconvolution algorithm[3], the deconvolved image is displayed in Suppl. Fig. 4.b.

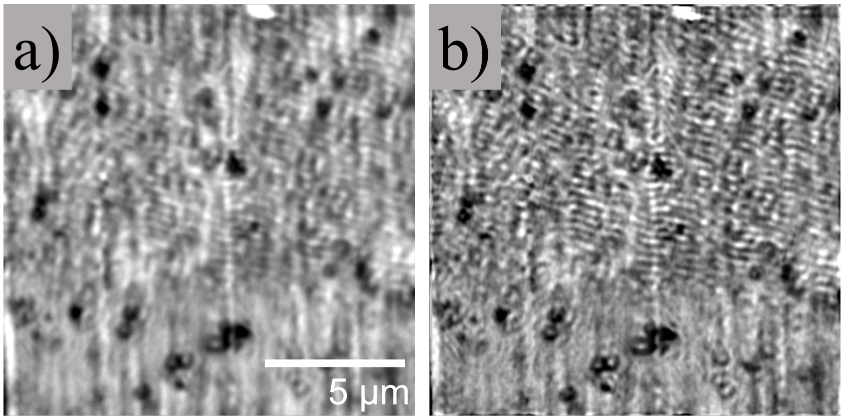

Figure 4. Re-scan CRM image deconvolution. a) Raw image, b) deconvolved image.

References

1. Bon, P., Maucort, G., Wattellier, B. & Monneret, S. Quadriwave lateral shearing interferometry for quantitative phase microscopy of living cells. *Opt. express* **17**, 13080–13094 (2009).

2. Schulz, O. *et al.* Resolution doubling in fluorescence microscopy with confocal spinning-disk image scanning microscopy. *Proc. Natl. Acad. Sci.* **110**, 21000–21005 (2013).

3. Ingaramo, M. *et al.* Richardson–lucy deconvolution as a general tool for combining images with complementary strengths. *ChemPhysChem* **15**, 794–800 (2014).
